# Properties of Gluten Intolerance: Gluten Structure, Evolution, Pathogenicity and Detoxification Capabilities

**DOI:** 10.3390/nu8100644

**Published:** 2016-10-18

**Authors:** Anastasia V. Balakireva, Andrey A. Zamyatnin

**Affiliations:** 1 Institute of Molecular Medicine, Sechenov First Moscow State Medical University, Moscow 119991, Russia; balakireva.anastacia@gmail.com; 2 Belozersky Institute of Physico-Chemical Biology, Lomonosov Moscow State University, Moscow 119992, Russia

**Keywords:** gluten, celiac disease, NCGS, wheat allergy, gluten intolerance, gliadin, glutenin, hordein, secalin, avenin

## Abstract

Theterm gluten intolerance may refer to three types of human disorders: autoimmune celiac disease (CD), allergy to wheat and non-celiac gluten sensitivity (NCGS). Gluten is a mixture of prolamin proteins present mostly in wheat, but also in barley, rye and oat. Gluten can be subdivided into three major groups: S-rich, S-poor and high molecular weight proteins. Prolamins within the groups possess similar structures and properties. All gluten proteins are evolutionarily connected and share the same ancestral origin. Gluten proteins are highly resistant to hydrolysis mediated by proteases of the human gastrointestinal tract. It results in emergence of pathogenic peptides, which cause CD and allergy in genetically predisposed people. There is a hierarchy of peptide toxicity and peptide recognition by T cells. Nowadays, there are several ways to detoxify gluten peptides: the most common is gluten-free diet (GFD), which has proved its effectiveness; prevention programs, enzymatic therapy, correction of gluten pathogenicity pathways and genetically modified grains with reduced immunotoxicity. A deep understanding of gluten intolerance underlying mechanisms and detailed knowledge of gluten properties may lead to the emergence of novel effective approaches for treatment of gluten-related disorders.

## 1. Introduction

Gluten intolerance is an umbrella term integrating three major types of gluten-related disorders: autoimmune celiac disease (CD), allergy to wheat and non-celiac gluten sensitivity (NCGS) [1,2,3]. Although these disorders possess similar symptoms, which include bloating, vomiting and diarrhea, a number of principle differences of their pathogenesis are remarkable (Table 1).

Celiac disease is an autoimmune enteropathy caused by genetic and environmental factors, with an estimated worldwide prevalence of about 1%. The huge prevalence of CD in the Saharawi people (5.6%) probably indicates that events linked to wheat domestication 10,000 years ago were a ‘founder effect’ related to the positive selection of HLA-DQ2 haplotype [4].

CD is usually diagnosed by serological examination [5]. Duodenal biopsy is not necessary for the diagnosis of CD but is necessary for the treatment [6]. Disease is induced by gluten-containing food in people carrying HLA-DQ2 or DQ8 haplotype (human leukocyte antigen Class II with DQ2 and/or DQ8 molecules on antigen-presenting cells). CD is not only characterized by gastrointestinal symptoms but also by extraintestinal manifestations, some of which are a direct consequence of autoimmunity responses—for example, dermatitis herpetiformis or gluten ataxia—while others are an indirect consequence of anaemia, such as osteoporosis, short stature and delayed puberty [7].

**Figure 1:**
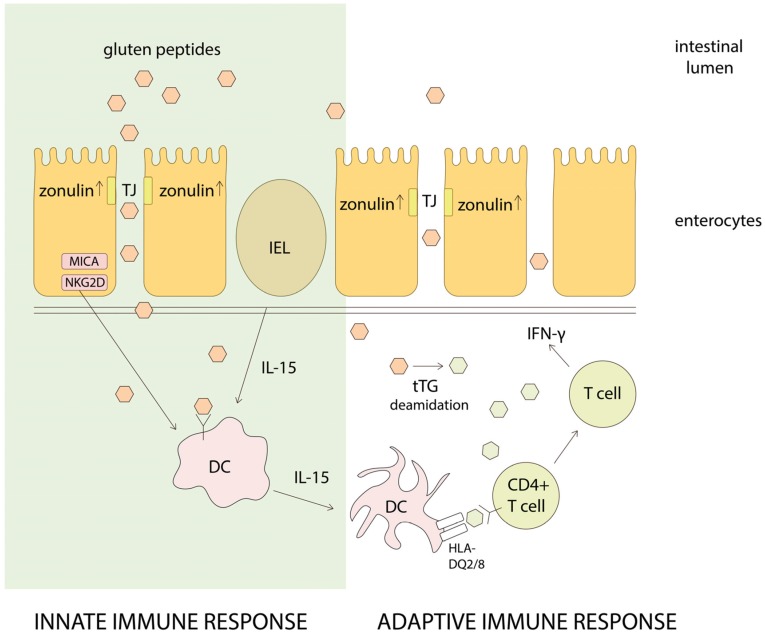
Schematic representation of major pathways in celiac disease (CD) pathogenesis. MICA, NKG2D—stress molecules on enterocytes, IEL—intraepithelial lymphocyte, DC—dendritic cell.

After gluten enters into the digestive system, glutamine and proline-rich gluten composing proteins are partially hydrolyzed by proteases presented in the gastrointestinal tract [8] (Figure 1). The upregulation of intestinal peptide zonulin, involved in tight junction regulation, appears to be partly responsible for the increased permeability characteristic of the gut [9]. As a result, generated gluten-derived peptides reach the lamina propria (mucosa) by transcellular or paracellular transport where they are modified by tissue transglutaminase (tTG) enhancing their affinity to MHC II molecules, and thereby making them toxic and immunogenic in HLA-DQ2 or DQ8 containing patients [10]. The repetitive presence of glutamine and proline residues determines the gluten-derived peptides as a preferred substrate for tTG. tTG-mediated modifications occur in two ways: deamidation (cleavage of the ε-amino group of a glutamine side chain) or more frequently transamidation (cross-linking of a glutamine residue from the gliadin peptide to a lysine residue of tTG). Further peptides presentation by HLA-DQ2/DQ8 protein subunits in the surface of dendritic cells to gluten-specific T cells induces two levels of immune response: the innate response and the adaptive (T-helper cell mediated) response with the production of interferon-γ and IL-15. As a result, it causes immune-mediated enteropathy, intestinal inflammation, followed by the atrophy of villi, crypt hyperplasia and increased infiltration by intraepithelial lymphocytes [11]. It also produces weight loss and chronic diarrhea. Although the causative agent is a dietary protein, the disease has marked autoimmune features, which are indicated by the presence of autoantibodies against tTG. Cross-linking between gliadin and tTG is covalent resulting in the formation of new epitopes, which trigger the primary immune response, and by which the autoantibodies against tTG are developed [12].

Allergy to wheat is represented by a food IgE-mediated allergy, which is most frequently based on the sensitization to wheat protein allergens. It has been shown that wheat ω5-gliadin is the main allergen of gluten, inducing wheat-dependent exercise-induced anaphylaxis [13]. Furthermore, some data suggest that α- and γ-gliadins are IgE-binding proteins [14]. Allergy occurs within a few hours and causes no permanent gastrointestinal or other organ damage. 

One more gluten-related disorder has recently been proposed—NCGS—and its pathogenesis is still not clear. Gluten ataxia (GA) is one of a number of different neurological manifestations attributed to CD, but Rodrigo et al. have suggested that it is related to NCGS [15]. Recently, special criteria aimed at optimizing the clinical care in clarifying the core of NCGS have been accepted [3].

Gluten triggers all kinds of gluten related disorders and represent proteins of wheat, barley, rye and, probably, oat. The gluten proteins of different species are the major subject of this present review along with the currently used proposed gluten detoxification strategies and the development of effective prevention and treatment of gluten related disorders.

## 2. Classification and Structure of Gluten Proteins

Gluten is a mixture of seed storage proteins found in grains such as wheat, rye, barley and oat. Wheat, rye and barley are closely related members of the *Triticeae* tribe. They contain kindred groups of proteins. Rye (*Secale cereal* L., genome composition RR) and barley (*Hordeum vulgare* L.) are diploid, while wheat is represented by the most widely studied hexaploid bread wheat (*Triticum aestivum* L., genome composition AABBDD), tetraploid pasta wheat (*Triticum durum* L., genome composition AABB) and diploid wheat (*Triticum monococcum* L., genome composition AA). Oat (*Avena sativa* L.) is the most closely related cereal to the *Triticeae* and belongs to a separate *Aveneae* tribe within the same sub-family (*Festucoideae*).

**Figure 2:**
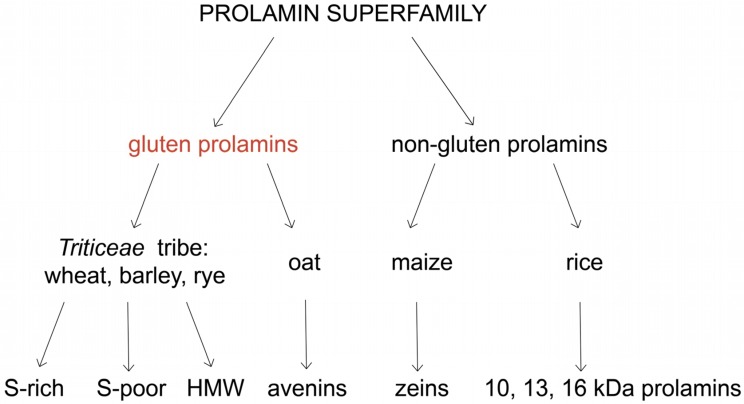
Prolamin Superfamily composition.

Gluten proteins appear to be prolamins due to the significant amount of glutamine and proline amino acid residues present in their primary structures. Prolamins are the major endosperm storage proteins in grains. Prolamin genes are present in the A, B and D genomes of wheat, and, consequently, hexaploid and tetraploid wheat prolamin fractions consist of more individual components than in barley and rye. There is also a difference in the number and properties of prolamin polypeptides. Despite these variations, all prolamins are related and, usually referred to as three broad groups: sulphur-rich (S-rich), sulphur-poor (S-poor) and high molecular weight (HMW) prolamins (Table 2) [16]. They comprise the Prolamin Superfamily, along with the prolamins of oat, maize and rice, (Figure 2).

**Figure 3:**
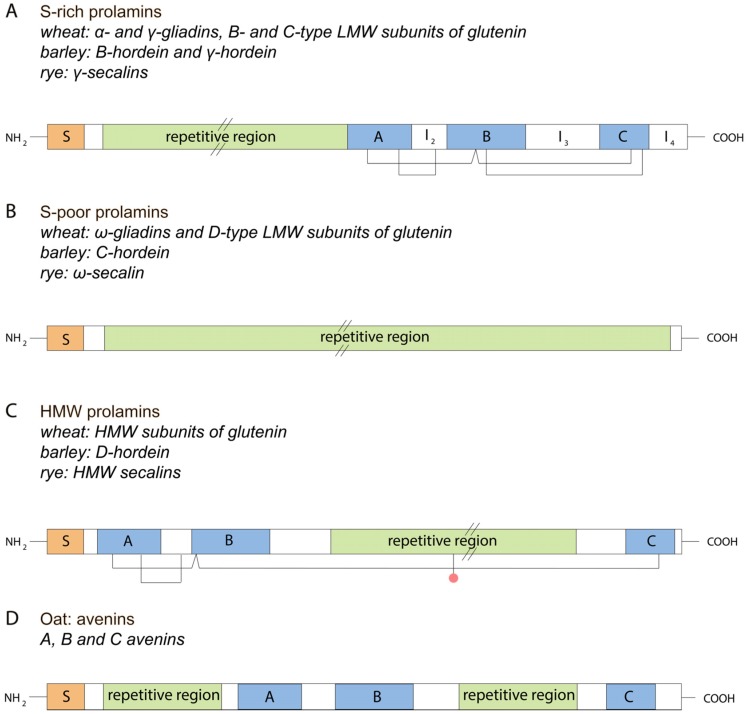
Schematic representation of typical structure of prolamin group members: S-rich, S-poor, HMW and avenins. S—signal peptide; A, B, C—conserved regions, lines—disulfide bonds, red circles—unpaired cysteine residue, I_2_–I_4_—variant regions; parallel lines—contracted repetitive region. (**A**) Typical structure of S-rich prolamin. It contains conservative domains, repetitive region and is able to form intrachain disulfide bonds; (**B**) Typical structure of S-poor prolamin. It lacks conservative domains and cysteine residues, and is therefore not able to form any disulfide bonds; (**C**) Typical structure of HMW prolamin. It contains conservative domains, repetitive region and is able to form intra- and interchain disulfide bonds; (**D**) Typical structure of avenin. It contains conservative domains, repetitive regions and is able to form interchain disulfide bonds only.

The proteins and polypeptides within these groups possess similar structures: signal peptide for translocation into cellular compartments, a non-repetitive *N*-terminal region, a non-repetitive *C*-terminal region and a long repetitive central region (Figure 3). The central region contains glutamine-rich and proline-rich repeat units unique to each group. It has been shown that the motifs in central region of S-rich and S-poor groups are clearly related and the cysteine positions in HMW proteins and S-rich group prolamins are highly conserved. Thus, the conclusion was that all these groups have a common evolutionary origin [17]. Now, we will discuss every prolamin group in detail.

### 2.1. Wheat

Wheat prolamins appear to be the first identified gluten proteins. According to their solubility they are usually divided into two classes: alcohol-soluble fraction named gliadins (monomeric) and insoluble—glutenins (polymeric, soluble in dilute acids and bases) [18]. It has been shown that gliadins contribute to the cohesiveness and extensibility of the gluten, whereas glutenins play a role in the maintenance of the elasticity and strength of the gluten [18]. Integrally, these proteins represent 80%–85% of gluten proteins and define viscoelastic properties of dough.

This difference in solubility largely reflects the ability of these proteins to form inter- or intramolecular disulfide bonds. Gliadins are monomeric proteins and are connected to each other through intrachain disulfide bonds (α/β-, and γ-gliadins), or not connected at all (ω-gliadins) [19]. It has been reported that C and D groups of LMW glutenin subunits (LMW-GS) are mainly composed of α-, β-, γ-, and ω-gliadins but mutated in cysteine residues. It means that LMW-GS can act as chain extenders depending on how many bonds it may form, and gliadins may serve as chain terminators [20]. The polymeric form contributes to the strength of gluten and improves dough quality.

(1) Gliadins

Gliadins are represented as single chain polypeptides, and it is accepted that gliadins are divided into four major groups (from fastest mobility to slowest): α-, β-, γ-, and ω-gliadins, according to their electrophoretic mobility in SDS-PAGE at low pH [18]. Precisely, ω-, α/β-, and γ-gliadins exist. Proteins from α- and β-groups are similar, so this group is referred to as α-gliadins [21]. ω-gliadins can be arranged into three types, which will be discussed further.

Genes encoding gliadin proteins are located on the short arms of Groups 1 and 6 chromosomes at three homologous loci—*Gli-A1*, *Gli-B1*, and *Gli-D1* (Group 1) and—*Gli-A2*, *Gli-B2*, and *Gli-D2* (Group 6) [22]. Some of the α- and γ-gliadins are encoded by *Gli-2* genes. The estimated copy number of α-gliadins in hexaploid wheat is between 25 and 150 copies. *Gli-1* contains genes encoding not only γ- and ω-gliadins but also LMW-GS, so there is a tight linkage between them [23].

Gliadins of different types bear distinct secondary structure. Thus, ω-gliadins contain randomly coiled β-turns without α-helices or β-sheets. In contrast, α/β- and γ-gliadins possess α-helices and β-sheets, which, in turn, allow these proteins to not only stabilize by disulfide bonds but also by the support of hydrogen bonds within their helices and sheets [24].

Gliadins are monomers but they are able to form intramolecular disulfide bonds. Free SH groups of glutenin, generated by β-elimination from cysteine, initiate SH–SS interchain reaction between gliadin and glutenin. This mechanism was proposed by Schofield et al. [25], which postulates that these SH–SS interchange reactions cause transformation from intra- to intermolecular SS bonds of gliadins [26]. Even the addition of free SH groups, such as cysteine, starts gliadin polymerization according to first-order reaction kinetics [27]. Such polymers are used as biodegradable films.

Gliadins are transported via the Golgi to the protein storage vacuole, whereas others, principally glutenins, are retained within the ER [16]. The precise mechanism determining the transportation of prolamins is not clear. There are no classical signal peptides targeting proteins neither to ER nor to vacuole.

(1.1)  α- and γ-gliadins

α- and γ-gliadins are very similar in their amino acid sequences. These types of proteins belong to S-rich group of prolamins and have similar structures (Figure 3). α- and γ-gliadins contain a relatively high composition of cysteine and methionine, but few glutamine, proline and phenylalanine residues. Eight cysteine residues allow the formation of intrachain disulphide bonds responsible for its folding (Figure 3) [28]. α- and γ-gliadins are able to form three and four intramolecular disulfide bonds, respectively. Their folded structures determine further non-covalent interactions, including hydrogen bonds and hydrophobic interactions [29].

*N*-terminal domain of α-gliadins consists of five residues and the central domain is about 113–134 amino acid residues. Central domain contains proline- and glutamine-rich heptapeptide PQPQPFP and pentapeptide PQQPY. This domain contains the most characteristic immunogenic fragment: 33-mer peptide comprising six overlapping epitopes significant for CD pathogenesis. Based on the differences in epitopes comprising 33-mer peptide, α-gliadins can be divided into six types. Only Type 1 encompasses proteins including 33-mer peptide (from hexaploid wheat), whereas other types do not [30].

*C*-terminal segment of α- and γ-gliadins is about 150 residues. In α-gliadins, almost all the glutamic acid and aspartic acid residues are present in amide forms [31]. γ-gliadins contain a 12 residues signal peptide and have more cysteine residues in their primary structure than α-gliadins. All these cysteine residues are involved in intrachain disulfide bonds formation (Figure 3).

Recently, a conformational equilibrium toward a beta-parallel structure was reported in the case of 33-mer peptide of α/β-gliadins under physiological conditions [32]. Gliadin nanoparticles formation was reported in distilled water (probably at pH 6–7) [33]. Then, self-organization capabilities of 33-mer peptide were investigated under gastrointestinal environment [34]. The spontaneous self-organization at pH 3.0 leads to the formation of aggregates such as micelles of amphiphilic molecules. Then, on increasing the pH to 7.0, gliadin nanostructures repulsion decreases due to proximity to the isoelectric point.

(1.2)  ω-gliadins

This group differs from other groups of gliadins. It is related to S-poor group of proteins lacking methionine or cysteine residues in their primary structure. Thus, ω-gliadins are incapable of disulfide bonds formation (Figure 3). As a result, no compact structure exists for these proteins. Proline, glutamine and phenylalanine residues comprise the majority of amino acids (80%) in ω-gliadins. They are more polar than α- and γ-gliadins [35].

On the basis of the *N*-terminal sequences, three different types of ω-gliadins have been distinguished from wheat proteins and related proteins from other species such as C-hordeins and ω-secalins: ARQ-, KEL-, and SRL-types depending on the first three amino acids [36]. The KEL-type differs from the ARQ-type by the absence of the first eight residues in its structure.

(2) Glutenins

Glutenins consist of subunits and are usually divided into two classes according to their molecular weight defined by SDS-PAGE: high molecular weight glutenin subunits (HMW-GS) and low molecular weight glutenin subunits (LMW-GS) (Table 2) [37]. They are encoded by *Glu-1* loci on the long arms of 1A, 1B, 1D chromosomes of wheat. 

Each HMW-GS locus contains two tightly linked genes encoding larger x-type (82–90 kDa) and smaller y-type (60–80 kDa) subunits, respectively [38]. Both types of subunits have similar structures (Figure 3). Repetitive central region is the cause of the difference between HMW-GS and LMW-GS. It may have various lengths provided by three types of repeat units: tripeptides (GQQ), hexapeptides (PGQGQQ), and nonapeptides (GYYPTSLQQ), and it is worth mentioning that the tripeptide units only exist in the x-type subunits, and both x- and y-type subunits possess hexapeptide and nonapeptide units [39]. The y-type glutenin subunits possess more cysteine residues than x-type subunits, and, are therefore capable of more inter- and intramolecular disulfide bonds formation, which mediates the aggregation of HMW-GSs with the involvement of LMW-GSs and results in an improved dough quality [16].

LMW-GS are classically subdivided into B, C, and D-type on the basis of their SDS-PAGE mobility and pI (Table 2) [40]. The B-type is the major group of LMW-GS. It represents the most numerous of LMW proteins. C-type proteins are the fastest moving type and the later discovered proteins comprise the D-type group. The B- and C-type subunits are encoded by genes located in the *Gli-3*, *Gli-1* and *Gli-2* loci on the short arm of homologous Groups 1 and 6 chromosomes. Genes at the *Gli-1* loci encode D subunits [41].

LMW-GS can be divided into two groups: one of which contains subunits with methionine as *N*-terminal amino acid (LMW-m) in their amino acid sequences, whereas the other group contains serine as *N*-terminal amino acid (LMW-s). In B- and C-types of LMW, there are both m- and s-types. D-type subunits are the less abundant group of LMW-GS. It has been shown that they could be formed by the mutation in one or more genes encoding ω-gliadins, resulting in the appearance of a single cysteine and allowing for the formation of an additional interchain disulfide bond in the glutenin macropolymer [42].

### 2.2. Barley

Hordeins are the major storage proteins in barley, and these proteins, like gliadins, are also alcohol-soluble prolamins and appear to be rich in glutamine and proline residues but poor in charged amino acids. Hordein polypeptides are not glycosylated. Two-dimensional polyacrylamide gel electrophoresis (with immobilized pH gradients in first dimension) of barley seed proteins reveal the occurrence of A, B, γ, C and D hordeins depending on their molecular mass and amino acid composition [43]. The A hordeins are of low molecular weight and do not seem to be true storage proteins. B and γ-hordeins are rich in sulfur and account for about 80% of the total hordein amount. They belong to S-rich prolamin group (Table 2, Figure 3); the C hordeins belong to S-poor group, and the D hordeins to HMW protein group. B and C hordeins collectively account for over 95% of barley seed storage proteins and are encoded by linked loci *Hor 2* (B hordein) and *Hor 1* (C hordein) located on the short arm of the chromosome 5 [44]. D hordeins and γ-hordeins are encoded by structural loci *Hor 3* and *Hor 5* located on the long arm of chromosome 5.

It has been suggested that B and C hordeins belong to common evolutionary origin due to the shared short tandem repeats [45]. The occurrence of two distinct domains in B hordein (one is related to C hordein and one is not) suggests that an unusual evolutionary pathway links these two groups of prolamin storage proteins.

The structural features of hordein proteins are similar to those of wheat proteins and are indicated in Figure 3. Three conserved regions (A, B, C) are present in all hordeins, except C hordein. These three regions also show a homology with each other, and contain cysteine residues that may be conserved within the groups or between the different groups of proteins. Shewry at al. concluded that S-poor prolamins originated from S-rich group because they have similar glutamine- and proline-rich motifs and evolved by the further amplification of repeat units and deletion on conserved regions A, B, and C [36].

(1) B and γ-hordein

B hordeins are the orthologous prolamin family to wheat LMW-GS group [17]. It has been estimated that B hordeins are represented by 11 different proteins and are now divided into closely related subgroups: SDS-PAGE revealed two major bands of B1 and B3 hordeins, and minor band with intermediate mass called B2. Three pseudogenes of B hordein have been identified [46]. Most of the B hordein are present in monomeric form or as single polypeptide subunits within the globules of low electron density of endosperm cells along with C, γ 1-, γ 2- and possibly γ 3-hordein polypeptides [47].

B hordeins can form a wedge or tadpole-shaped structure stabilized with interchain disulphide bonds formed between unpaired cysteine residues in the *N*- and *C*-terminal domains [48].

**Figure 4:**
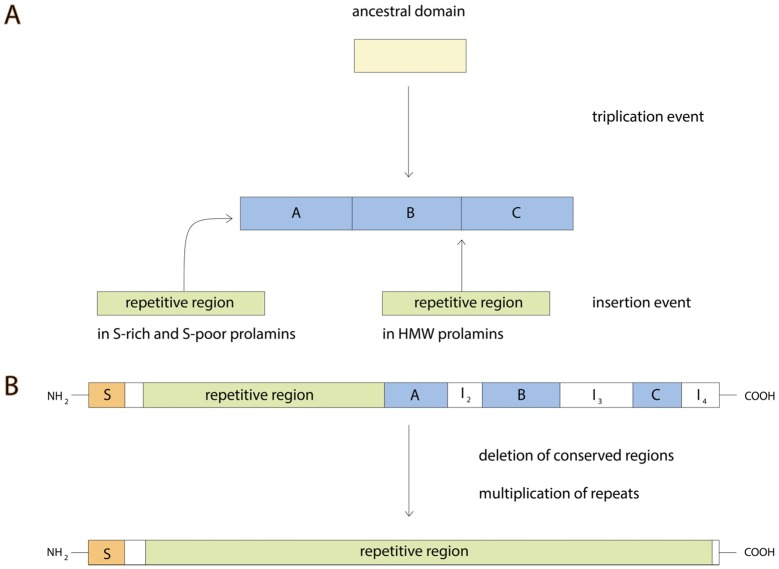
Summary of evolutionary events that probably contributed to the divergence of Prolamin Superfamily proteins. A, B, C—conserved regions, S—signal peptide, I_2_–I_4_—variant regions. (**A**) Conservative domains A, B and C of prolamins are thought to originate from the ancestral domain by triplication. S-rich and HMW prolamins emerged after insertions of repetitive regions in a manner showed on a Figure; (**B**) S-poor prolamins are suggested to originate from S-rich prolamins by deletion of conserved A, B and C regions, and by multiplication of repeated sequences.

γ-hordein is homologues to γ-gliadin of wheat. γ-hordein is presented in γ1-, γ2-, γ3-types. Analysis of primary sequences revealed a distant relation between γ3-hordein to γ2- and B hordein, while γ2-hordein is very close to γ-gliadin and γ-secalin (Figure 4) [47]. In addition, γ1- and γ2-types have identical *N*-terminal sequences. Signal peptides allow γ1-, γ2-hordeins to be co-translationally transported into the rough endoplasmic reticulum. They present in small aggregates (hordein polypeptides) soluble in 55% isopropanol. γ1- and γ2-hordein can form intermolecular disulfide bridges but γ3-hordein exists as a monomer only.

(2) C hordein

C hordein is a group of homologous proteins that have molecular weights of about 50 kDa. C1 and C2 types of C hordein were identified [49]. They are homologous to wheat ω-gliadin. C hordeins lack cysteine residues and always present in monomeric form due to their inability to form disulfide bonds. Their primary sequences are rich in glutamine, proline and phenylalanine residues. They possess short *N*- and *C*-terminal (unique sequence of 6 amino acid residues) domains and central domain containing P/LQQPY and PQQPFPQQ repetitive motifs (Figure 3) [45]. Structural studies of C hordein showed that these proteins have a conserved but unusual secondary structure—repetitive β-turns [50]. Further analysis performed by l’Anson e al. indicates that such primary structure results in a similarly conserved supersecondary structure called “worm-like” chain. This is a loose spiral based on elements of P-turn and poly-l-proline II helix [51]. C hordeins are located within the globules of low electron density along with γ-hordein and B hordein [47]. These globules merge with each other in cytoplasm.

(3) D hordein

D hordein is homologous to HMW glutenins of wheat. They have been studied in detail due to their importance in the quality and strength of dough. D hordein and polymeric B hordein are present in polymeric form as aggregates of polypeptide subunits linked by interchain disulfide bonds. It is always deposited in the vacuole [52]. D hordein possesses a similar amino acid composition as HMW-GSs of wheat. It has repeat units such as tripeptides (GQQ), hexapeptides (PGQGQQ), and nonapeptides (GYYPTSLQQ). These subunits form spiral supersecondary structure provided by repeating β-turns [53]. Nevertheless, it has unique tetrapeptide present in *C*-terminal part of repetitive domain. D hordein has an extended rod-like structure. In addition, D hordein differs from HMW-GS in terms of the number and distribution of cysteine residues.

### 2.3. Rye

Rye is one of the major cereal species along with wheat and barley. Prolamins of rye are called secalins and are divided into three classes: HMW secalins, γ-secalins and ω-secalins (Table 2) [17].

(1) HMW secalin subunits (HMW-SS)

These high molecular weight proteins are encoded by two genes of *Sec-3 (Glu-R1)* locus located at the long arm of 1R rye chromosome [54]. As HMW glutenins are subdivided into x- and y-types, *Sec-3* also consists of two paralogous alleles (*Glu-R1x* and *Glu-R1y*) of duplication origin. They encode x-(more abundant) and y-types of subunits [55]. HMW-SS are always present as one or two individual subunits similar to D hordeins and in contrast to five to six subunits of wheat HMW-GS.

HMW-SS are homologous to HMW-GS but there is a significant difference in the properties and structural parameters determining gluten formation (see below, Triticale).

Repetitive domain of HMW-SS contains tripeptide, nonapeptide and hexapeptide consensus motifs discussed in Section (2) Glutenin section. Scanning tunnelling microscopy of a purified HMW secalin subunit demonstrated aligned rods with a diameter of about 1.9 nm containing diagonal striations (presumably corresponding to turns of the spiral) and having a pitch of about 1.5 nm [56].

(2) γ-secalins

γ-secalins are encoded by five to 10 genes of *Sec-1* and *Sec-2* loci at 1R and 2R chromosomes of rye, respectively. The structures of γ-secalin and other γ-type prolamins are alike (Figure 3). It has eight cysteine residues involved in intramolecular disulfide bonds formation and unpaired cysteine residue involved in intermolecular bonds formation. Rye also encodes 75 kDa γ-secalins that have no analogues in other cereals. It amounts to about 50% of total seed proteins in rye and is sometimes separated from other secalins into distinct types.

(3) ω-secalins

*Sec-1* locus is a gene region of ω-secalins located at the short arm of chromosome 1RS. This arm contains *Sec-1* disease resistant genes tightly linked to leaf, stem and stripe rusts and powdery mildew [57]. This linkage results in some dough quality defects such as marked stickiness, reduced strength and intolerance to overmixing. Clarke et al. reported that the *Sec-1* locus of rye consists of approximately 145 kb of DNA containing a tandem gene array of 15 ω-secalin gene units [58]. FISH analysis shows that the sizes of the *Sec-1* region range from 131 to 164 kb on the DNA fiber specimen [59]. Rye genome contains not only ω-secalin genes with ORFs but also pseudogenes, which may be the subject of a reduced selection pressure [60].

ω-secalins are related to S-poor group of prolamins and possess a typical structure for this group. These proteins are monomers and cannot form interchain disulfide bonds like the other proteins in this group: C-hordeins and ω-gliadins. This was discussed earlier for C hordein, ω-secalin has no A, B or C conservative domains. Repetitive domain of ω-secalin is flanked by short unique sequences of *N*-terminal 12 amino acid residues and four amino acids on its *C*-terminus. Repetitive region of ω-secalin also has an unusual supersecondary structure similar to that in C hordein of barley.

### 2.4. Triticale

Hybrid species triticale (X *Triticosecale Wittmack*) also contains gluten, and originates from species durum wheat (*Triticum durum* L., AABB genome) and rye (*Secale cereal* L., RR genome). The hexaploid triticale genome (AABBRR) encodes three sets of HMW glutenins (1A and 1B chromosomes), HMW secalins (1R), 75K γ-secalins (2R) of rye and LMW glutenins (1A and 1B). This complex was named “secaloglutenin”, while “secalogluten” refers to the hydrated network of secaloglutenin with some monomers [61]. This network is weak and incohesive and the dough strength is between the dough strength of *Triticum durum* L. and *Secale cereale* L. and requires less mixing time. Currently, many papers focus on the elucidation of possible methods for the improvement of triticale dough.

### 2.5. Oat

Prolamins in oat (*Avena sativa* L.) are represented by avenins. Oat avenins differ from other grain prolamins in the lower amount of proline. Furthermore, oats contains a relatively low content of storage proteins; approximately 10% only of the total grain protein amount compared with 40%–50% in wheat, barley, and rye [62]. This is a cause of the inability to divide oat prolamins into HMW proteins, S-rich and S-poor groups in a manner of *Triticeae* tribe. Avenins were shown to be homologous to α/β-gliadin and γ-gliadin of wheat, B-hordein of barley and γ-secalin of rye (S-rich group) [63]. 

Avenins can be well analyzed with HPLC technique, and contain insoluble and soluble fractions. Insoluble in alcohol but soluble in reducing solution fraction named “glutelin fraction” [64]. The molecular weight of avenins is about 18.5–23.5 kDa and contain two blocks of glutamine- and proline-rich repeated sequences, whose length varies from six to 11 residues (Figure 3). Avenins are monomers and only contain interchain disulfide bonds [62].

Although avenins are very similar [65], only differing by point mutations, they are subdivided into A, B and C groups according to the neighbor-joining phylogeny method [66]. Avenin sequences belonging to B and C groups possess eight cysteine residues, whereas sequences from A group bear 9. Thus, avenins from group A are capable of interchain disulfide bonds formation and a polymer in a wheat glutenin manner with a use of unpaired nineth cysteine. Generated polymer may consist of only A avenins or from other prolamins resulting in heteropolymer formation. It has been proposed that A avenins are LMW-GS-like (glutelin fraction). B and C avenins show up to be α- and γ-gliadins-like proteins. The expression levels of avenins of different groups have not yet been well studied, but it is clear that α- and γ-gliadins-like proteins (group C and B) expression is greater than that of LMW-GS-like avenins from group A [66]. Avenins are synthesized and assembled into vacuolar protein bodies in developing endosperm tissue along with globulin storage proteins. Immunogold staining of this tissue demonstrated that prolamins were located in the light-staining regions. These proteins appear to aggregate within the rough ER, while most of the globulin appear to aggregate in the vacuole [67].

## 3. Gluten Evolution

Although the prolamin superfamily seems to be a relatively unique group of proteins, there is evidence of a relationship between these proteins and other seed protein groups. First, proteins within one group (S-rich, S-poor or HMW) have a similar structure. For example, comparison of prolamin *C*-terminal domain sequences from S-rich group of proteins (wheat, barley and rye) including oat avenins showed significant similarity. Particularly, three conserved regions of length about 30 residues were identified and called A, B and C (Figure 3) [17]. They include a conserved number and position of cysteine residues. It is interesting that these regions share some similarity indicating the probable triplication of a short ancestral sequence (Figure 4) [62]. Moreover, such short similar sequences were found not only in gluten prolamins but also in other seed and non-seed proteins.

Comparison of regions of A, B and C conserved domains (Figure 3, see I_2_–I_4_) identified subgroups within S-rich group: α-type and γ-type, B hordein [68]. γ-type is considered to be the most ancient among the gluten proteins. It is worth mentioning that regions A, B and C are also present in the HMW prolamins, although in this case regions A and B are located within the *N*-terminal domain while region C is within the *C*-terminal domain indicating that proteins of S-rich and HMW groups arose from a single ancestor by insertion of I_2_–I_4_ blocks and repeated sequences. It has been shown that α- and γ-gliadins are both related to LMW-GS. Moreover, it has been suggested that C and D groups of glutenins are mainly composed of α-, β-, γ-, and ω-gliadins but mutated in cysteine residues [20].

Proteins of S-poor group of prolamins do not contain conserved regions. It is also clear that repeated sequences of S-poor prolamins (ω-type, C hordein) are related to repeated sequences in S-rich prolamins indicating that S-poor group of proteins originated from S-rich group through amplification of repeats and deletion of *C*-terminal domain. The evolutionary events leading to emergence of prolamin superfamily are summarized in Figure 4.

## 4. Gluten Intolerance Pathogenesis

### 4.1. Cross-Reactivity between Gluten Proteins

Primarily, gluten is a source of flour and, consequently, bread and flour products. Consumption of gluten-containing food makes such food an immune system target. Digested gluten is a reason for the emergence of different antigens and immunogens. Cross-reactivity implies the reaction between an antibody and an antigen that differs from the immunogen, and it has been shown in glutenin-specific and gliadin-specific T-cells. Such T-cells could respond to gliadin and glutenin and vice versa due to their directivity to repetitive sequence highly homologous in these proteins [69]. Such cross-reactivity contributes to the development and spread of T-cell response and inflammation. It is also known that CD is characterized not only by inflammation and small intestine tissue remodeling but also by neurologic defects such as axonal neuropathy and cerebellar ataxia [70,71]. It has been shown that such neurological implications may occur partly due to the cross-reactivity between antigliadin antibody and synapsin I protein [72]. This protein is a cytosolic phosphoprotein found in most neurons of the central and peripheral nervous systems. Although gliadins are not homologous to synapsin I, there is a glutamine- and proline-rich-region in *C*-terminal sequence of synapsin I, which includes PQP and PQQP motifs similar to those in gliadin.

Evidence of the cross-reactivity of prolamin proteins was also reported in the course of allergy. ω5-gliadin is the major wheat allergen. It was shown that anti-ω5-gliadin antibodies bind to rye γ70-secalin, rye γ35-secalin and barley γ3-hordein [73]. In about 90% of patients with wheat-dependent exercise-induced anaphylaxis, IgE antibodies against these proteins were found. Rye γ70- and γ35-secalin and barley γ3-hordein cross-react with ω5-gliadin. This probably happens due to the fact that IgE antibodies bind to structurally similar epitopes; found proteins are related to the same evolutionary group of γ-type prolamins.

Even though there is no cross-reactivity between allergens in oat and other gluten species, avenins show immunological cross-reactivity to γ-secalin due to their considerable homology [74]. First immunogenic peptides in hordein, secalin and avenin were revealed on the basis of T-cell cross-reactivity against wheat gluten proteins [75]. Epitopes defined in hordein and secalin were recognized by α-gliadin-reactive T-cell lines in vitro while avenin epitopes were not. That explains why rye and barley were considered to be pathogenic for CD patients, whereas oat was included in the “gluten-free” group of food.

Later, it was shown that oat consumption is safe for the majority of CD patients [76], even for children [77]. It is well known that the greater the proline residues in storage protein the more pathogenic this protein is for CD patients [78]. Low proline content may be the reason why oat avenins are less immunogenic compared to wheat prolamins but may still be toxic in large quantities. However, there was no direct (in vivo) evidence of the activation of gluten-reactive T-cells following ingestion of oats. Hardy et al. provided in vivo evidence that ingestion of oats activates avenin-specific T-cells in 10% of CD patients [79]. Moreover, they showed T-cells to be cross-reactive against hordein and avenin. After oral challenge with barley (and not wheat or rye) the majority of HLA-DQ2.5 CD patients harbor T-cells capable of being activated by avenin peptides ex vivo, but the ingestion of oats itself provides rather weak antigenic stimulation for this population of T-cells. Avenins are probably less stimulatory because they do not contain proteolytically resistant peptides longer that 10 amino acid residues. They have reduced binding stability to HLA-DQ2.5 compared to hordein peptides. This means that avenin-reactive T-cells are activated by the consumption of barley rather than oat.

### 4.2. Celiac Disease

As described in the introduction, gluten is impregnable by the gastric, pancreatic and intestinal digestive proteases of people carrying HLA-DQ2 or/and DQ8 haplotype. HLA-DQ is a part of the MHC class II antigen-presenting receptor system and distinguishes its own and foreign cells. HLA-DQ protein consists of two subunits, which are encoded by the HLA-DQA1 and HLA-DQB1 genes located on the short arm of the 6 chromosome. Mainly, people with celiac disease have DQ2 or DQ8 isoforms because these receptors bind to gliadin peptides more tightly than other forms.

**Figure 5:**
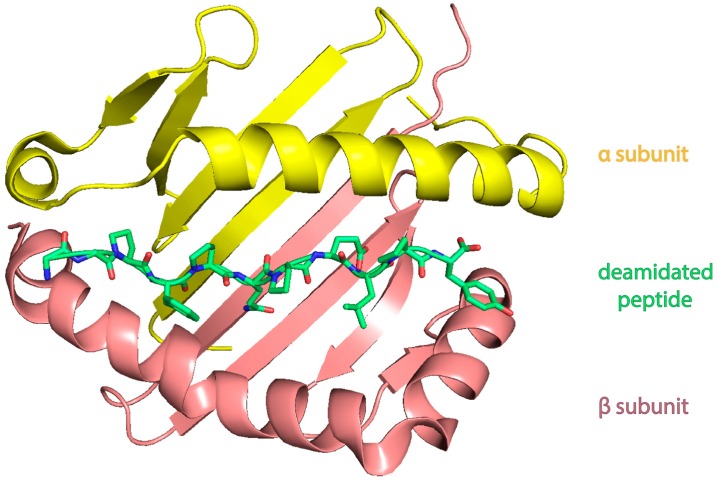
3D reconstruction of DQ α^5^-β^2^-binding cleft with a deamidated α-gliadin peptide (green), using PyMOL (PBD ID 1S9V) [81].

However, there are multiple DQ2 haplotypes. The most associated with celiac disease (95% of patients) is the two-gene HLA-DQ2 haplotype referred to as DQ2.5. This haplotype is composed of subunits α^5^ and β^2^ encoded by two adjacent gene alleles DQA1*0501 and DQB1*0201 (Figure 5). Four percent of CD patients have the DQ2.2 isoform (DQA1*0201:DQB1*0202) and the remaining have DQ8 (encoded by the haplotype DQA1*03:DQB1*0302).

After the gluten enters into the digestive system, prolamin proteins are not fully hydrolyzed by proteases, which results in the emergence of gluten peptides. They are deamidated by tTG enhancing their affinity to MHC II molecules. Deamidated peptide is then recognized by DQ molecule on the surface of a dendritic cell and is presented to T cells inducing immune response. It is interesting that both DQ2 and DQ8 lack canonical aspartic acid residue at DQβ57. It results in the compensation of this negative charge by negatively charged residues either in the T cell receptor or in the deamidated peptide. Absence of aspartic acid residue leads to cross-reactive and stronger responses by T cells [80].

Nowadays, it is clear that distinct gluten peptides are involved in celiac disease in a different manner. Peptides are divided into two groups: toxic and immunogenic. Toxic peptides are capable of inducing mucosal damage when administered in vivo on the intestine, whereas peptide is considered to be immunogenic if it is able to specifically stimulate HLA-DQ-restricted T cell lines isolated from peripheral blood of CD patients [82]. Peptides differ from each other in the degree of the immunogenicity. It is remarkable that some are immunodominant, meaning that they evoke strong T cell response in almost every CD patient, whereas the immunogenic do not. Immunogenicity is enhanced after tTG deamidation procedure. It is worth mentioning that tTG is more likely activated after inflammation, but it is still not clear whether the deamidation of peptides is by tTG initiates inflammation or vice versa [82].

All gluten proteins (gliadin and glutenin from wheat, hordein from barley, secalin from rye and avenin from oat) possess their own sets of toxic and immunogenic peptides (or epitopes) with distinct immunogenicities. However, gliadin peptides are known to be the most toxic and numerous, specifically derived from α- and γ-gliadin: the strongest and most common adaptive response to gluten is directed toward an α2-gliadin fragment of 33 amino acids in length [8]. Digestion of gliadin results in the emergence of two pieces: 25-mer (p31-55, it can be degraded to smaller peptides) and 33-mer (p57-89). Peptide p31-43 of α2-gliadin may directly induce interleukin-15 production from enterocytes and dendritic cells. Peptide p57-89 is deamidated by tTG and presented to T cells by HLA-DQ molecules. Glutenin peptides are also involved in T cell response [83]. Peptides may enter the cell by endocytosis, with their entrance into the cells requiring 37 °C temperature and Ca^2+^ in the media [84]. It has been shown that these peptides possess structural configuration characterized by a left-handed polyproline II helical conformation that is preferred by MHC class II ligands [85].

(1) Properties of 33-mer peptide from α-gliadin

**Figure 6:**
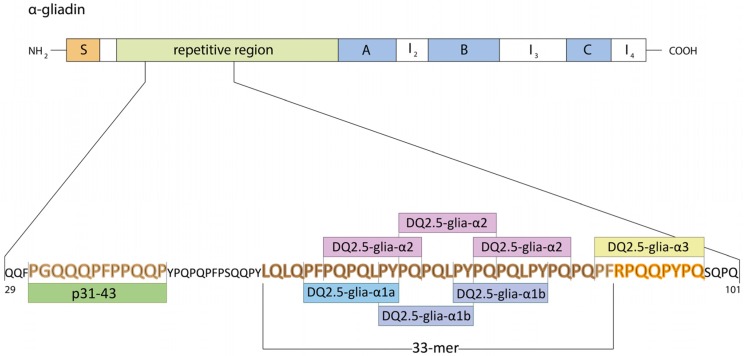
Fragment of α-gliadin protein sequence. Main immunogenic fragments: peptides p31-43, 33-mer and DQ2.5-glia-α3 are indicated.

The α-gliadin 33-mer is one of the digestion-resistant gluten peptides that is highly reactive to isolated celiac T cells and is the main immunodominant toxic peptide in celiac patients. It is located in the *N*-terminal repetitive region of α-gliadin and contains six overlapping copies of three different DQ2-restricted epitopes (Figure 6) [86].

Using RNA-amplicon sequencing (NGS) technology it was shown that α-gliadins can be separated into six types and only one type contains all the immunogenic peptides and epitopes, whereas the other five types do not contain all the epitopes disabling 33-mer peptide formation [30]. Thus, distinct types of α-gliadins differ mainly in the number of repeat blocks consisting in interspersed motifs PFPPQQ and PYPQPQ.

Six epitopes of type 1 α-gliadin are DQ2.5-glia-α1a/b and DQ2.5-glia-α2 (Figure 6). There is also a partial overlap with 33-mer DQ2.5-glia-α3 epitope associated only with type 1 of gliadins. 33-mer is able to self-assemble in a concentration-dependent manner through structural transition [32]. It obtains polyproline II structure based on type II beta-turn with increase of peptide concentration.

33-mer reaches lamina propria and after deamidation plays a central role in the pathogenic cascade of celiac disease by activating the adaptive immune response. 33-mer enters the cell by intracellular pathway, excluding paracellular entrance. Gliadin-derived peptides can also be transcytosed from the apical of the intestinal epithelium to the basolateral side along with transferrin and IgA, avoiding entrance to the late endocytic compartment [87].

In vivo experiments revealed that 33-mer gliadin-derived peptide is undigested by enzymes of the intestinal brush border. Moreover, in a monkey model of gluten sensitivity, 33-mer peptide can be detected in the serum when the disease starts, indicating that this peptide can trespass the mucosa intact in vivo [88].

Then, 33-mer is deamidated by tTG present in the intestinal brush border and presented by dendritic cell to T cell (in mesenteric lymph nodes). T cells reach peripheral blood through the thoracic duct and product interferon-γ resulting in intestine epithelial cytotoxicity, while another peptide p31-43 has been reported to induce the innate immune response necessary to initiate the T-cell adaptive response through production of interleukin-15.

It has been shown that 33-mer of α-gliadin is very similar to protein Prn of *B. pertussis*, which causes pertussis. These results show that neither pertussis immunization nor disease induces production of antibodies reactive against the peptide, and thus it is unlikely that either pertussis immunization or disease contributes to CD pathogenesis on the basis of cross-reactive antibodies [89].

(2) Repertoire of gluten peptides active in CD

It has been established that deamidated forms of gluten peptides are more toxic than their amidated forms. tTG preferably deamidates sites QXP (X—any amino acid residue), which are abundant in immunodominant peptides. Interestingly, both DQ2 and DQ8 molecules lack the aspartic acid residue at β57 position present in other DQ molecules. DQ2 and DQ8 molecules possess positively charged pockets containing five anchor positions and a least three of them (P4, P6, and P7) prefer to bind negatively charged amino acids [90]. Crystallographic structure of DQ2 complexes with immunodominant epitope revealed that glutamic acid residue fits in the P4, P6 and P7 anchor positions and proline residue—in the P1, P3, P6 and P8 positions [91]. Analogous report regarding DQ8 complexes revealed only two glutamic acid-preferred positions (P1 and P9). This explains the lower number of gluten peptides active in DQ8 individuals.

Long-term T cell lines (TCL) or T cell clones (TCC) raised against gluten are used to identifying gluten immunogenic peptides. Anderson et al. established an approach that detects gluten-specific T cells in the peripheral blood (peripheral blood mononuclear cells, PBMC) after 3 days of consumption of gluten-containing food by an interferon-γ EliSpot assay [92]. A comprehensive, quantitative mapping of T cell epitopes was used to screen all the unique 20-mer sequences of gliadins, glutenins, hordeins, and secalins. Independently, this screening and EliSpot assay provided the set of immunodominant epitopes from wheat, barley, and rye (Table 3) [93,94]. Almost all DQ2 immunogenic peptides of α-gliadins map the *N*-terminal 57–89 region (33-mer). DQ2-restricted ω-gliadin peptides strictly related to α-gliadin 17-mer: it contains two overlapping copies of 9-mer epitopes. Immunogenic peptides of γ-gliadins are spread along all the sequences. Similarly, few DQ2-restricted sequences from secalin and hordein proteins were reported to stimulate intestinal CD4+ T cell lines or clones [75].

Only 3 DQ8-restricted epitopes were identified using T cell lines or T cell clones: two for α-gliadin, γ-gliadin and one for glutenin [95]. Additional peptides were discovered as a result of the work by Tye-Din et al. [93]. Furthermore, it was shown that HLA-DQ8-associated CD appears not to be exclusively dependent on deamidation by tTG [95]. 

Avenins differ from other groups of prolamins due to their low content of proline and glutamine residues. Nevertheless, a few gliadin-like and glutenin-like avenin-derived peptides were identified [96]. Avenin peptides were divided into three groups: low-stimulatory short peptides (six residues), stimulatory (27 and 10 residues) and peptides with upregulated stimulatory capacity (10 and 14 residues). Larger peptides (27 residues) are commensurable in size with 33-mer and induce response of dendritic cells in not only CD patients but also in control healthy patients. Whereas peptides with the appropriate size and disposition of amino acids residues (10 and 14 residues) are likely to go through a differential endocytic pathway. 

Thus, immunogenic sequences were identified in all gluten proteins of *Triticeae* and oat. These studies revealed that amongst all the DQ2-restricted peptides of wheat, barley, rye and oat prolamins, there is a hierarchy of T cell recognition depending on the specific cereal ingested. Furthermore, an evident redundancy in DQ2-restricted peptide recognition occurs, i.e., activated by a dominant peptide T cells are capable of recognizing and responding to a large number of related gluten sequences and vice versa. However, there is no clear difference in the immunogenicity strength between DQ8-restricted peptides. Altogether, DQ8-type of HLA molecules are less strongly associated with celiac disease, compared to DQ2. Oat peptides possess the lowest immunogenic activity though avenin peptides are capable of inducing T cell response.

### 4.3. Wheat Allergy

Wheat flour triggers IgE-mediated food allergy and is one of the top eight food allergens. Wheat allergy commonly develops in childhood [97]. When an allergen specifically binds to IgE antibodies, it induces the activation of mast cells and basophils. In the case of wheat, it is believed that allergy occurs due to a breach in oral tolerance and as a consequence of Th2-biased immune dysregulation that induces sensitization and B-cell-specific allergen IgE production [98]. Gluten proteins causing allergy include some types of ω-gliadin as well as non-gluten protein of wheat such as profilin, serpin, α-purothionin, etc. Non-gluten flour proteins and some γ-, α/β-gliadins can cause an occupational respiratory allergy such as baker’s asthma, which appears after the inhalation of flour by millers or bakers. ω5-gliadins trigger another type of allergy—food allergy—referred to as wheat-dependent exercise-induced anaphylaxis (WDEIA), which develops after the ingestion of wheat followed by intense physical exercise.

Recently, it was shown that γ-, α/β-, ω5-, ω1,2-gliadins contain IgE-binding epitopes as well as HMW and LMW subunits of glutenin [99] (Table 4). Nevertheless, major allergenic protein of wheat is ω5-gliadin possessing *N*-terminal sequence SRLL, which can be crucial for allergy pathogenesis, and repetitive region consists almost entirely of peptides FPQQQ and QQIPQQ [14].

γ-gliadin, α/β-gliadin and ω1,2-gliadin are causative allergens in both WDEIA patients and those with baker's asthma [100,101]. Epitopes QQPFP and PQQPF of gliadin are also involved in atopic dermatitis-related wheat allergy [102] as well as QQQPP motif in LMW-GS [103].

Nowadays, 3D structure of known IgE allergenic epitopes helps to elucidate its conformation and to produce recombinant allergens for further research.

### 4.4. Non-Celiac Gluten Sensitivity (NCGS)

The first reported NCGS cases were described as longstanding and previously unresolved history of abdominal pain, discomfort, bloating, altered bowel habit and fatigue with exclusion of celiac disease. NCGS is more frequently diagnosed in adults rather than in children [104]. In most cases, NCGS reveals itself a few hours after gluten digestion [104]. Similarly to CD patients, patients with NCGS suffer from nutritional deficiencies, coexisting autoimmunity, and a decreased bone mineral density compared with the general population. The prevalence of HLA-DQ2 and/or HLA-DQ8 genotypes is ~50% in NCGS comparable to the general population [105], but there is no anti-tTG2 antibodies identified. Gluten only triggered an innate immune response in NCGS and provoked an additional adaptive immune response with increased expression of IL-6, IL-21, IL-17 and IFN-γ [106]. However, gastrointestinal symptoms other than intestinal permeability and adaptive immune responses are not involved in the process. In NCGS, gliadins do not induce mucosal inflammation in vitro or the activation of basophils as seen in CD [107].

It has been suggested that in NCGS gluten-related peptides enter the systemic circulation and cause extraintestinal manifestations such as ataxia, neuropathy and encephalopathy [108]. Moreover, it has been proposed that gluten causes depression, anxiety, autism and schizophrenia in patients with NCGS [109], and also reported that psychosis might be a manifestation of NCGS [110].

Nowadays, gluten-related disorders have often been recognized as commonly mimicking irritable bowel syndrome (IBS) because of the similar symptoms such as abdominal pain, bloating, bowel habit abnormalities (either diarrhea or constipation) [111]. Indeed, both can coexist independently without necessarily sharing a common pathophysiological basis. Furthermore, the microbiome may also play a role in the pathogenesis of NCGS [112]. Gut microbiota composition and metabolomic profiles may influence the loss of gluten tolerance and subsequent onset of gluten intolerance in genetically-susceptible individuals [113]. Gut microbiota could become a target for further therapy [114].

Recently, a standardizing protocol was reported for the diagnostic confirmation of NCGS [3]. It implies assessment of the clinical response to GFD and consequent effect of the gluten challenge. It is important that patients are on a normal, gluten-containing diet for proper evaluation, which is not always possible. For these two assessment steps, a modified version of the Gastrointestinal Symptom Rating Scale (GSRS) is used. The GSRS protocol is based on reviews and the clinical experience and allows for evaluation of gastrointestinal and extra-intestinal symptoms. Patients name one to three symptoms and the Numerical Rating Scale (NRS) measures the severity score from 1 to 10. However, there are still difficulties in diagnosing and managing NCGS. Even though the precise mechanism and biochemical markers for the NCGS disease have still not identified, this protocol could be used to establish the prevalence of this condition.

## 5. Gluten Detoxification Strategies

### 5.1. Gluten Free Diet (GFD)

There is currently only one proven effective way of treating celiac disease and NCGS—a gluten free diet. It means the avoidance of gluten-containing food in gluten intolerance patients’ ration. There is little information in the literature on minimal disease-eliciting doses of gluten, which would be safe for CD patients. Apparently, it should lie between 10 and 50–100 mg daily intake [115]. Starch-based gluten-free products contain trace amounts of gluten. However, a diet completely devoid of gluten is unrealistic. The diet is complicated due to cross-contamination and/or the presence of small amounts of the gluten in food and medicines.

GFD cannot be regarded as a healthy diet. Gluten-free products are usually made with starches or refined flours characterized by low fiber content. It is known that the consumption of adequate amounts of dietary fiber is related to important health benefits such as prevention of colon cancer, diabetes and cardiovascular disease [116]. Thus, GFD may lead to possible nutrient deficiencies in fiber resulting in consequent diseases. Several studies suggest using pseudo-cereal sources of fiber instead of gluten-free products in order to maintain the necessary fiber content level [117].

GFD also leads to deficiency in Vitamins C, B12, D and folic acid [118], which is associated not only with malabsorption caused by villi atrophy but also with low quality of GFD [117]. Consuming fruits and vegetables rich in vitamins and antioxidant substances up to five times a day is recommended. Some studies demonstrate that gluten-free cereal products contain lower amounts of folate compared to their gluten-containing counterparts, so there is a need for additional folate supplementation [119].

In CD patients, malabsorption and inflammation contribute to a low bone mineral density (BMD) [120]. CD patients have a 40% higher risk of having bone fractures compared with non-CD healthy people. Thus, the diet plays a critical role in the maintenance of proper bone mineralization. GFD appears to be unbalanced in terms of calcium, magnesium, zinc in male and iron in women, and additional supplementation required [121]. Zinc is an essential trace element involved in numerous reactions and biochemical functions. Zinc deficiency can affect protein synthesis and leads to growth arrest [122]. Magnesium is essential for several enzymatic reactions (for DNA and RNA polymerases, ionic pumps and calcium channels). Thus, it is recommended that gluten-free products are substituted with other cereals such as quinoa, sorghum and amaranth, which are safe and rich in folic acid, vitamins (riboflavin, Vitamin C and Vitamin E) and minerals [117].

At the same time, gluten-free diet contains high amounts of sugar and hydrogenated fats, which could result in the occurrence of hyperinsulinemia and an increased obesity risk [123].

Thus, GFD appears to be an unbalanced diet inadequate in terms of both macro- and micronutrients. In order to maintain the necessary level of all the nutrients an annual screening for nutrient status of a patient is required and there is a need for additional nutrient supplementation. Thus, GFD is not an optimal and healthy way to treat all the manifestations of gluten intolerance including CD, wheat allergy and NCGS. Even though a wheat free diet is optimal for wheat allergy treatment, patients can eat rye, barley and oat. A wheat free diet is also probably effective for NCGS treatment. Other directions of treatment of gluten intolerance need to be developed.

Many works focus on providing new medicinal approaches for effective gluten intolerance treatment. Two major directions exist: prevention and treatment of gluten related disorders. The prevention hypothesis implies that the time the gluten is introduced into the diet of infants at risk of CD may affect the disease incidence. The Prevent CD Family Study was held in 10 European countries. One thousand children and their mothers participated, and were followed up for a period of 1–3 years. It has been suggested that small quantities of gluten are administered gradually to induce oral immune tolerance to gluten. It is now accepted that gluten may be introduced into the infant’s diet at any time between 4 and 12 months of age. In children at high risk of CD, an earlier introduction of gluten (4 vs. 6 months or 6 vs. 12 months) is associated with an earlier development of CD, but the cumulative incidence in later childhood is similar [124]. Recently, an analogous program in Italy was started to evaluate the at-risk infants age, at which CD-related autoimmune serological changes occur. Data obtained in this study indicate that delaying the gluten introduction into the infants’ diet until the age of 12 months decreases the prevalence of CD [125]. Both studies need a much longer follow-up analysis to establish whether the timing of gluten exposure can really prevent CD or merely delay its onset.

### 5.2. Detoxification of Gluten Proteins with Enzymatic Therapy

This approach is based on the fact that gluten peptides are highly resistant to digestive pancreatic and brush border proteases. Fortunately, many organisms (e.g., bacteria, fungi, plants etc.) encode proteolytic enzymes possessing distinct features compared to endogenous proteases presented in human [126,127,128]. Thus, it has been proposed that exogenous enzymes can be employed for additional enzyme supplement therapy to promote the complete digestion of cereal proteins, and thus destroy T-cell gluten epitopes, in particular [129,130]. A number of peptidases possessing glutenase activities were isolated from germinating cereals (*Hordeum vulgare* L., *Triticum aestivum* L.), bacteria (*Flavobacterium meningosepticum*, *Sphingomonas capsulate*, *Myxococcus xanthus*), fungi (*Aspergillus niger*, *Aspergillus oryzae*), and stored-product pest yellow mealworm (*Tenebrio molitor*) [131,132,133,134,135]. One of them is ALV003 enzyme—modified recombinant EP-B2 enzyme from barley, and prolyl endopeptidase from bacteria *Sphingomonas capsulate*—was shown to be effective in vitro and in vivo, non-toxic and without allergic reactions [136,137]. Gluten-containing food can also be treated with bacterial-derived peptidases, in particular, proteases of certain lactobacilli present in sourdough are able to proteolyze proline-rich gluten peptides [138].

### 5.3. Modified Grains

There are several studies targeted at developing grains with reduced pathogenicity. On the basis of knowledge of peptide immunogenicity hierarchy, site-directed mutagenesis of wheat, which would not affect the baking properties, has also been proposed. However, hexaploidity of wheat seriously complicates this process. Nevertheless, successful transformation of bread wheat *Triticum aestivum* Butte 86 was reported [139]. In this paper, a subclass of ω-gliadins genes, encoding proteins that cause food IgE-mediated allergy, were silenced in order to decrease the level of ω5-gliadins in grain. Transgenic wheat has reduced allergenicity without influencing the dough quality. Similar work was performed to reduce the toxicity in CD patients of all gliadin proteins through the shutdown of these genes by RNA interference [140]. Genes of γ-, α- and ω-gliadins were down-regulated in these plant lines. This has led to the production of wheat lines with very low levels of toxicity for CD patients. 

As discussed above, the gluten of barley and rye is also highly pathogenic for patients with gluten intolerance. Thus, a number of works describe modifications introduced into barley genome: for example, deletion of B and C hordeins resulted in 20-fold reduced immunotoxicity compared to wild type [141].

Nowadays, the wheat genome is modified in order to improve the dough quality. However, different modifications may introduce known or clinically cross-reactive allergens into genome. It was suggested that bioinformatic methods can be used to prevent such allergen introduction and assess the safety and allergenicity of modified crops, using a comprehensive database [142].

### 5.4. Corrections of Gluten Pathogenicity Pathways

tTG is very important in CD pathogenesis. For this reason, it has become a target for suppression by the design of potent and selective inhibitors. Inhibition of tTG2 by cystamine in vitro and in situ was confirmed by means of abolished reactivity of gliadin-specific T-cell response [143]. Recently, Keillor et al. reviewed the latest and most applicable inhibitors of tTG2 designed on the basis of the conformational effects and crystallographic structures of inhibited tTG2 [144].

Zonulin, one of the TJ regulatory proteins involved in the proper functioning of intestinal epithelial permeability, controls the passage through the mucosal barrier. The inhibition of zonulin overexpression can prevent it trespassing the gut barrier. The effective synthetic peptide inhibitor was developed and named as AT1001 or Larazotide acetate [145]. There is now a novel therapeutic agent targeting TJ regulation in patients with CD.

Peptides themselves are undoubtedly major CD participants. Peptide analogues of gliadin epitopes can be engineered with antagonistic effects of native peptides. Nexvax2^®^ (Immusan T, Inc., Cambridge, MA, USA) is the peptide-based therapeutic vaccine based on desensitization therapy principles [146]. This product encompasses three peptides that respond to a substantial proportion of the T-cell reaction to gluten in HLA-DQ2-carring patients. Nexvax2 is currently undergoing clinical trials.

## 6. Conclusions

Nowadays, gluten intolerance is an important issue. The number of people diagnosed with gluten intolerance is increasing. Thus, there is a need for more effective and novel approaches to treat gluten-related disorders. Externally, it is caused by the consumption of gluten prolamin proteins present in wheat, barley and rye. In the present paper, we have summarized the knowledge on the classification, properties, structure, evolution and role of gluten proteins in the pathogenesis of gluten intolerance manifestations. Even though gluten proteins—gliadins, glutenins, hordeins, secalins and avenins—share similar features and evolutional origins, they possess different pathogenicities. A detailed understanding of the principal properties of gluten intolerance causative agents open ups the possibilities for the development of novel therapeutic approaches such as with improved low pathogenic wheat, barley and rye plant lines; renewed therapeutic enzymatic drugs and vaccines. This will obviate the need for GFD and improve the quality of life of people suffering from gluten intolerance.

## Figures and Tables

**Table 1:** Comparative major characteristics of gluten intolerance manifestations.

	Celiac Disease	Allergy	NCGS
Underlying cause	Genetic: HLA-DQ2 or/and –DQ8 haplotype	Atopy (100%)	Probably, genetic: DQ2 and/or DQ8 (up to 50% of patients)
Laboratory markers	IgA (IgG) anti-tTG, IgA(IgG) anti-endomysium (anti-EMA), anti-deamidated gliadin peptides antibodies	Specific IgE for wheat, specific IgE for ω-5 gliadin, specific IgE for non-specific lipid transfer proteins	IgG antigliadin antibodies (in only a part of the patients)
Histopathological intestine symptoms	Atrophy of villi, crypt hyperplasia, increased infiltration by intraepithelial lymphocytes	Any mucosal damage or increased infiltration by intraepithelial lymphocytes or atrophy of villi and crypt hyperplasia	Any mucosal damage or increased infiltration by intraepithelial lymphocytes

**Table 2:** Classification of gluten prolamins.

Grain Species	Components	Molecular Weight (% Total)	Polymers or Monomers
**HMW Prolamins**			
Wheat	HMW subunits of glutenin	65–90 kDa (6%–10%)	Polymers
Barley	D-hordeins	>100 kDa (2%–4%)	Polymers
Rye	HMW secalins	>100 kDa (2%)	Polymers
**S-rich prolamins**			
Wheat	γ-gliadins	30–45 kDa (70%–80%)	Monomers
	α-gliadins		Monomers
	B- and C-type LMW subunits of glutenin		Polymers
Barley	B-hordeins and γ-hordeins	32–45 kDa (80%)	Aggregated type, monomers or single chain polypeptide
Rye	γ-secalins	40–75 kDa (80%)	Polymers
**S-poor prolamins**			
Wheat	ω-gliadins	30–75 kDa (10%–20%)	Monomers
	D-type LMW subunits of glutenin		Aggregated type, polymers
Barley	C-hordeins	40–72 kDa (10%–15%)	Monomers
Rye	ω-secalins	48–55 kDa (10%–15%)	Monomers
**Other gluten prolamins**			
Oat	avenins	18.5–23.5 kDa (10%)	Monomers

**Table 3:** Number of gluten immunogenic peptides currently identified within distinct gluten proteins.

Grain Species	Gluten Protein	Number of DQ2-Restricted Peptides Identified (Confirmed in Vitro on TCLs/TCCs or/and on PBMCs after in Vivo Challenge)	Number of DQ8-Restricted Peptides Identified (Confirmed in Vitro on TCLs/TCCs or/and on PBMCs after in Vivo Challenge)
Wheat	α-gliadins	3	3
	γ-gliadins	11	4
	ω-gliadins	3	4
	Glutenins	3	1
Barley	Hordeins	8	-
Rye	Secalins	11	-
Oat	Avenins	6	-

**Table 4:** Currently identified IgE-binding epitopes in wheat gluten proteins. *—X—any amino acid.

Protein	IgE-Binding Epitope Motifs
α/β-gliadin	QQQFPGQQ, LQQQ
γ-gliadin	QPQQPFPQ
ω5-gliadin	QQXPXQQ *
ω1,2-gliadin	QQPXPXQ
HMW-GS	QQPGQ(GQQ)
LMW-GS	QQPIQQQP

## Abbreviations

CD	celiac disease
NCGS	non-celiac gluten sensitivity
HLA	human leukocyte antigen
MHC	major histocompatibility complex
tTG	tissue transglutaminase
IgA (IgG, IgE)	immunoglobulin A (G, E)
IEL	intraepithelial lymphocyte
TJ	tight junction
DC	dendritic cell
GA	gluten ataxia
HMW	high molecular weight
LMW	low molecular weight
HMW-GS	high molecular weight glutenin subunits
LMW-GS	low molecular weight glutenin subunits
HMW-SS	high molecular weight secalin subunits
SDS-PAGE	sodium dodecyl sulfate polyacrylamide gel electrophoresis
ER	endoplasmic reticulum
DNA	deoxyribonucleic acid
RNA	ribonucleic acid
FISH	fluorescence in situ hybridization
HPLC	high-performance liquid chromatography
PDB	protein data bank
NGS	next-generation sequencing
IFN-γ	interferon-γ
IL	interleukin
TCL	T cell line
TCC	T cell clone
PBMC	peripheral blood mononuclear cells
WDEIA	wheat-dependent exercise-induced anaphylaxis
GFD	gluten-free diet
IBS	irritable bowel syndrome
GSRS	gastrointestinal symptom rating scale
NRS	numerical rating scale
BMD	bone mineral density
